# Evaluating Diagnostic Tests With Near-Perfect Specificity: Use of a Hui–Walter Approach When Designing a Trial of a DIVA Test for Bovine Tuberculosis

**DOI:** 10.3389/fvets.2018.00192

**Published:** 2018-08-15

**Authors:** Gustaf Rydevik, Giles T. Innocent, Iain J. McKendrick

**Affiliations:** ^1^Roslin Institute, The University of Edinburgh, Edinburgh, United Kingdom; ^2^Biomathematics and Statistics Scotland, Edinburgh, United Kingdom

**Keywords:** non-gold standard test estimation, Hui–Walter, disease eradication, rare disease surveillance, bovine tuberculosis

## Abstract

Active surveillance of rare infectious diseases requires diagnostic tests to have high specificity, otherwise the false positive results can outnumber the true cases detected, leading to low positive predictive values. Where a positive result can have economic consequences, such as the cull of a bovine Tuberculosis (bTB) positive herd, establishing a high specificity becomes particularly important. When evaluating new diagnostic tests against a “gold standard” reference test with assumed perfect sensitivity and specificity, calculation of sample sizes are commonly done using a normal approximation to the binomial distribution, although this approach can be misleading. As the expected specificity of the evaluated diagnostic test nears 100%, the errors arising from this approximation are appreciable. Alternatively, it is straightforward to calculate the sample size by using more appropriate confidence intervals, while precisely quantifying the effect of sampling variability using the binomial distribution. However, regardless of the approach, if specificity is high the sample size required becomes large, and the gold standard may be prohibitively costly. An alternative to a gold standard test is to use at least two imperfect, conditionally independent tests, and to analyse the results using a variant of the approach initially proposed by Hui and Walter. We show how this method performs for tests with near-perfect specificity; in particular we show that the sample size required to deliver useful bounds on the precision becomes very large for both approaches. We illustrate these concepts using simulation studies carried out to support the design of a trial of a bTB vaccine and a diagnostic that is able to “Differentiate Infected and Vaccinated Animals” (DIVA). Both test characteristics and the efficacy of the bTB vaccine will influence the sample size required for the study. We propose an improved methodology using a two stage approach to evaluating diagnostic tests in low disease prevalence populations. By combining an initial gold standard pilot study with a larger study analyzed using a Hui–Walter approach, the sample size required for each study can be reduced and the precision of the specificity estimate improved, since information from both studies is combined.

## 1. Introduction

Diagnostic testing plays a crucial role in the surveillance and detection of infectious diseases. In its simplest form, we take a sample from an individual, and use our test, for example an antibody test, to say whether the individual is infected or not. As all tests are imperfect, knowing the behavior of the diagnostic tests used for surveillance is fundamental in order to evaluate collected surveillance data; of particular importance are the reliability and error rates of the tests used.

Classically, diagnostic tests have been treated as having a binary response, producing either a positive or a negative result. The diagnostic test gives a result that can be classified as “positive” if the individual is infected, and a result that can be classified as “negative” if the individual is not infected. Denote by *D*_+_ (*D*_−_) an infected (non-infected) individual, and *t*_+_ (*t*_−_) a positive (negative) test result. The probability that a test of an infected animal produces a positive result is commonly referred to as the *sensitivity* of the test, *Se* = *P*(*t*_+_|*D*_+_), and the probability that a test of a non-infected animal produces a negative result, *Sp* = *P*(*t*_−_|*D*_−_), is commonly referred to as the *specificity* of the test. This terminology was first introduced in 1961 by Thorner and Remein (1) in a US Department of Health publication. Together, sensitivity and specificity fully describe the expected behavior of a binary test, when used in a population and on an infection where both sensitivity and specificity can be assumed to be the same for all individuals.

In order to estimate the specificity of a test, the ideal situation is to test a population that is known to be free from disease, and produce statistical estimates and confidence intervals of the specificity from the number of negative test results relative to the number of individuals in the population. Similarly, in order to evaluate the sensitivity of a test, the ideal situation is to test a population where all individuals are known to be diseased, and estimate the sensitivity from the proportion of positive test results. However, such an ideal situation is rare, as it requires use of either a perfect test to pre-screen the individuals, or a controlled infection study. It is also problematic that, in the latter situation, results may not be representative of the test responses likely to be seen in field use.

A somewhat more realistic approach is to test a population with unknown prevalence with two tests; one reference test where the sensitivity and specificity is already known, and another test that is being evaluated. In such a situation, it is possible to estimate the unknown sensitivity and specificity of the new test. However, this creates a “Catch-22” situation, as the properties of the reference test will have needed to have been evaluated at some point in the past.

A statistical method for estimating the properties of two diagnostic tests at once was pioneered by Hui and Walter (2). In this seminal paper, they showed that it is possible to estimate the unknown sensitivity and specificity of two different diagnostic tests simultaneously, if both tests are used on all individuals in two populations with different, potentially unknown, prevalences of infection. The approach used in their paper is a variant of “Latent Class Analysis,” a widely used approach in modern statistical science 3.

In the UK and elsewhere, bovine tuberculosis (bTB) is of political, public health and economic interest (4). Eradication using a test-and-cull policy made good progress during the 1960s, 70s, and the early 1980s. However, certain areas of England and Wales still have ongoing issues with high levels of infection in cattle herds (5, 6). One possible response to this situation would be to vaccinate cattle against bTB, a strategy first suggested in the 1940s (7). However, in order subsequently to demonstrate freedom from disease it would be necessary to develop and validate a diagnostic test able to Distinguish Infected and Vaccinated Animals (DIVA). Such a DIVA test has been developed at the UK Animal and Plant Health Agency (APHA) (8) using two independent panels of antigens. We assume that the results from these two panels will be conditionally independent. This test has not been fully evaluated in terms of its sensitivity and specificity. These properties are important in determining whether or not the use of a test is viable in practice. In order for this test to be cost effective in use in the UK situation, the work of Conlan et al. (9) indicates that the specificity needs to be as high as 99.85%.

Here, we develop the latent class approach in a situation where a vaccine can be expected to interfere with diagnostic test performance. The assumptions are that two populations with different prevalences of disease are available, that each population is divided into one known vaccinated and one known unvaccinated subpopulation, and two diagnostic tests are used on all four sub-populations. We allow sensitivity and specificity to vary for vaccinated and unvaccinated individuals. It is demonstrated that it is possible to extend the latent class framework to provide an estimate of the unknown vaccine efficacy in addition to the unknown parameters (sensitivity and specificity) for both tests, and the prevalences of disease in the populations. The new model framework is applied to a trial of a DIVA test for bTB, being used to estimate the effect of study size on estimates of test sensitivity and specificity.

## 2. Methods

### 2.1. Comparison of methods to calculate the sample size for gold-standard diagnostic test evaluation

If there exists a test that has a one-to-one relationship with reality, i.e., if the test results indicate that an individual is positive, then that animal is truly infected, and conversely that if the results indicate that the individual is negative then it truly is uninfected, then that test is referred to as a “gold standard” test.

Determining test sensitivity and specificity in the presence of a gold standard test is relatively simple. Using the gold standard test, identify a group of positive animals and a group of negative animals. The proportion of the positive animals that test positive is then our estimate of the sensitivity, the proportion of negative animals that test negative is the estimate of specificity. If the event that an animal is tested can be thought of as a random sample from the population of all possible animals with the same disease status (either negative or positive), and that we can assume that across each population all animals have the same probability of being tested as positive, then the number of test positives from a sample of animals will be distributed as a binomial random variable.

Formal assessment of test properties can be formulated as an assessment of whether the confidence intervals of the sensitivity and specificity contain values which users would regard as unacceptable. Given assumptions about the true sensitivity and specificity, in turn, such assessments can be quantified as questions related to the width of the estimated confidence interval. Where estimators are consistent, the width of the confidence interval will reduce as the sample size increases, and studies can be powered so as to give rise to estimates with the required precision. In practice, different approaches can be taken to the calculation of the confidence interval around a binomial proportion. Provided the sample size is sufficiently large, it is common to assume that the proportion is distributed as a Gaussian random variable with mean $\hat{p}$ and variance $\hat{p}\left(1-\hat{p}\right)/n$, where $\hat{p}=y/n$, the ratio of the number of successes *y*, to the total number of trials *n*, and to use the Normal approximation to the binomial to construct a 95% confidence interval (CI). However, whilst this approximation works well when $\hat{p}$ is around 0.5, it will be poor when $\hat{p}$ is close to zero or one. Alternative methods exist to estimate confidence intervals in situations where the Normal approximation is not valid. For example, in a Bayesian framework we can examine the posterior of the appropriate beta distribution to provide estimates of median, mean and mode for the proportion, along with credibility intervals. However, a straightforward approach to implement, with conservative coverage properties, is the Clopper–Pearson “exact” method described in Collett (10). If we denote the lower and upper limits of the 100(1−α)% CI as *p*_*l*_ and *p*_*u*_, then these will be consistent with the following equalities:

$$\begin{array}{l} \overset{j=0}{\underset{y}{\sum^{\text{​}}}}\left(\frac{n}{j}\right)p_{u}^{j}{\left(1-p_{u}\right)}^{n-j}=\frac{\alpha }{2}\\ \overset{j=y}{\underset{n}{\sum^{\text{​}}}}\left(\frac{n}{j}\right)p_{l}^{j}{\left(1-p_{l}\right)}^{n-j}=\frac{\alpha }{2}, \end{array}$$

which can be solved to give:

$$\begin{array}{l} p_{l}\left(y,n\right)=\frac{y}{y+\left(n-y+1\right)F_{2\left(n-y+1\right),2y}\left(\alpha /2\right)}\\ p_{u}\left(y,n\right)=\frac{y+1}{y+1+\left(n-y\right)/F_{2\left(y+1\right),2\left(n-y\right)}\left(\alpha /2\right)}, \end{array}$$

where *F*_*a, b*_(*p*) is the upper (100p)% point of the *F* distribution with *a, b* degrees of freedom.

The approach taken to calculate sample sizes should be consistent with the approach which it is anticipated will be used to calculate confidence intervals. A commonly used approach is presented in Thrusfield (11), where the equation for the CI based on the normal approximation is inverted to provide an estimate of the *n* required to achieve a precision of ±ψ%. We will refer to this as the “Approximate” method. This approach neglects the sampling variability intrinsic to all experiments or studies, assuming that the observed sensitivity or specificity will exactly match the true (assumed) value *p*. In making this assumption, this approach is guaranteed to underestimate the sample sizes required to achieve any specified precision. It is straightforward to develop an approach to sample size calculation which makes use of an exact confidence interval, and which also allows for sampling error. For any given *n* (number of animals) we identify the set of possible observed values

$$\begin{array}{l} Y=\left\{y:p_{u}-p_{l}<2\psi \right\}\cap \left\{y:p\in \left(p_{u},p_{l}\right)\right\}, \end{array}$$

i.e., the set of outcomes for which, on average, the estimated confidence interval will have a precision no worse than ±ψ%, and will contain the assumed true value. Calculating

$$\begin{array}{l} P(\text{Success};n)=\underset{y\in Y}{\sum^{\text{​}}}\left(\frac{n}{y}\right)p^{y}{\left(1-p\right)}^{n-y}, \end{array}$$

and repeating for increasing values of *n*, we seek to identify the smallest value of *n* for which *P*(Success;*n*)≥ a specified quantity, which we will set equal to 80%. This is the probability of “success” in observing a sufficiently precise confidence interval. We will refer to this as the “Exact” method.

### 2.2. An adaption of the hui–walter paradigm for DIVA tests

If we have two diagnostic tests where the probability of a positive result for one test carried out on a positive animal is independent of the probability of the animal being positive on the second test, and both probabilities depend only on the sensitivity of the relevant test, then by using the laws of independent probabilities the probability that a positive animal is observed as positive to both, test 1 only, test 2 only or neither test is *Se*_1_×*Se*_2_, *Se*_1_×(1−*Se*_2_), (1−*Se*_1_) × *Se*_2_ and (1−*Se*_1_) × (1−*Se*_2_), respectively.

Within a single population we can then infer the probability that an animal of unknown status has one of the four possible test outcomes if we use the population prevalence. This allows us to define a set of equations for the probability of the four possible outcomes as follows:

$$\begin{array}{lll} Pr\left(++|pop_{1}\right) & = & Se_{1}\times Se_{2}\times p_{1}+\left(1-Sp_{1}\right)\;\times \left(1-Sp_{2}\right)\\ \times \;\left(1-p_{1}\right)\\ Pr\left(+-|pop_{1}\right) & = & Se_{1}\times \left(1-Se_{2}\right)\times p_{1}+\left(1-Sp_{1}\right)\times Sp_{2}\\ \;\times \;\left(1-p_{1}\right)\\ Pr\left(-+|pop_{1}\right) & = & \left(1-Se_{1}\right)\times Se_{2}\times p_{1}+Sp_{1}\times \left(1-Sp_{2}\right)\\ \times \;\left(1-p_{1}\right)\\ Pr\left(--|pop_{1}\right) & = & \left(1-Se_{1}\right)\;\times \left(1-Se_{2}\right)\times p_{1}+Sp_{1}\times Sp_{2}\\ \times \;\left(1-p_{1}\right). \end{array}$$

In these equations, *Pr*(++|*pop*_1_) is the probability that both tests are positive for an animal in population 1, *Se*_1_ is the sensitivity of test 1, *Sp*_1_ is the specificity of test 1, *p*_1_ is the proportion of animals in population 1 that are truly positive, and other parameters can be defined by analogy. Hence, the numbers positive to both tests, positive to the first only, to the second test only and positive to neither test will follow a multinomial distribution with these probabilities. Given that we know the total numbers of animals tested within the population, the test result observations from each population provide us with three degrees of freedom for estimation.

If we have a second, independent population we can construct similar probabilities for population 2. With these quantities we can infer estimates for all sensitivities, specificities and prevalences (six unknowns in total). In order to ensure that the system is identifiable, that is that a unique solution to the equations is possible, it is necessary to ensure that the prevalence in the two populations is different, i.e., have a high prevalence and a low prevalence population.

We have extended this approach, originally described by Hui and Walter (2), to account for vaccination. Each test now has two associated sensitivities and two specificities: those for vaccinated animals and those for unvaccinated. In order to solve the new equations, and to be able to infer estimates for the entire set of four sensitivities and four specificities we require four populations, giving rise to 12 independent equations. These can be produced by analogy, by taking any three of the equations above and substituting *pop*_2_, *pop*_3_, or *pop*_4_ for *pop*_1_, and *Se*_3_, *Se*_4_, *Sp*_3_, or *Sp*_4_ for *Se*_1_, *Se*_2_, *Sp*_1_, and *Sp*_2_ appropriately. This is simplified since two of the populations are now vaccinated, and two unvaccinated, and this status is known. If the prevalences in the multiple underlying populations are sufficiently different, then we can calculate estimates of all four sensitivities, four specificities, and four prevalences. Via a slight re-parameterization and by making an additional assumption of equal vaccine efficacy across the underlying populations, we can replace the need for four prevalence parameters with two prevalence parameters and a single value for the vaccine efficacy. We set *p*_1_ = *e* × *p*_2_ and *p*_3_ = *e* × *p*_4_, where *p*_1_ is the prevalence in the vaccinated, high prevalence population, *p*_2_ the prevalence in the unvaccinated, high prevalence population, and *e* the reduction in prevalence due to vaccination: which we define as the vaccine efficacy. The prevalences *p*_3_ and *p*_4_ are defined similarly in the low prevalence population. This approach has the advantage of allowing a simple estimate of vaccine efficacy also to be estimated. However, it should be noted that although this estimate has epidemiological interpretation it is neither the focus of this method, nor is it the usual value taken as the vaccine efficacy.

### 2.3. Data simulation and analysis

Data were simulated in R (12) and Bayesian-Markov Chain Monte Carlo (MCMC) analyses were performed in JAGS (13). Parameter values are given in Table 1. In some analyses, which we refer to as latent class analyses, a relatively small number of animals are tested by both the gold standard and DIVA tests, i.e., the true animal status is known. In analyses that we refer to as combined analyses a larger number of animals are tested by both tests. The results from this part of the simulation are used to provide priors to the analysis in the absence of the gold standard test, i.e., the majority of animals.

**Table 1:** Table of the various parameters, and the values used in the simulation model.

**Parameter**	**Values used**
Number of simulations per scenario	1,000
Total number of animals sampled	10,000–100,000 in steps of 10,000
Ratio of animals in four prevalence-vaccine groups	1:1:1:1
Prevalence in low prevalence population	0.05
Prevalence in high prevalence population	0.20
Sensitivity of both tests in all population	0.75
Specificity of both tests in all populations	0.999 or 0.995
Vaccine efficacy in all populations	0.6
Number of positive animals identified using a gold standard test	30 or 300
Number of negative animals identified using a gold standard test	100 or 1,000

*Where more than one value is given all possible combinations with all other parameter values were simulated*.

For each simulation, data for each combined test result in each population are simulated from a multinomial distribution with probabilities as specified in section 2.2 above. The resulting pseudo-data are saved to a file and passed to JAGS along with a description of the likelihood in the JAGS language which uses the same equations to infer parameters from the simulated data. We use minimally informative, uniform priors for all parameters, except that the results of the simulated gold standard comparison are used to produce beta distributions for the test sensitivities and specificities. That is, if the simulation of 1,000 negative animals results in *y* positive tests from the DIVA test, then the prior used for the test specificity would be a beta(100-*y*+1, *y*+1) distribution. Following burn-in on five chains, each was sampled for 1,000 iterations which were combined to produce a 5,000 iteration posterior sample.

In this application we are not particularly interested in the estimates for prevalence or vaccine efficacy, but rather in the sensitivity and specificity of the two tests. For these we construct 95% CIs by selecting the 2.5th and 97.5th percentiles, that is, the values ranked 125th and 4,875th of the 5,000 iterations used to define the posterior distribution.

If two populations with different prevalences are not available, then it is possible to use the standard Hui–Walter model, and consider vaccinated animals as a separate population to unvaccinated animals, with a lower prevalence. However, in such an analysis it is necessary to assume that the sensitivity and specificity are the same for vaccinated and unvaccinated animals, which may not be a reasonable assumption in practice.

Although no formal definition of power can be formulated within a Bayesian framework, we can extend the framework already presented for the exact method. In this application, we are particularly interested in the test specificity. In our case we assume that although both tests are used in the test evaluation, an outcome where only one is confirmed as having a sufficiently high specificity will be satisfactory. We want any study to be able to determine that the specificity of a single test is >0.9985, as that is the minimum value predicted by Conlan et al. (9) as being financially viable in practice. We therefore define power as the proportion of simulated data sets for a given set of parameters for which the 2.5th percentile of the posterior distribution for the specificity for at least one of the tests is at least 0.9985.

### 2.4. Application to bTB DIVA test evaluation

For bTB there is no true gold standard test. It is generally accepted that post-mortem identification of lesions typical of infection, and subsequent identification of the organism, is proof that an animal is infected. However, the converse cannot be said to be true: early in infection lesions may be small or non-existent, and even late in infection lesions may be small and few in number in some individuals. Hence, genuinely infected animals may be misclassified (14). In addition, although positive animals are truly positive, they do not represent the whole population of positive animals; there will be a sub-population of positive animals with either lesions too small to find, or no lesions at all. Using a latent class approach allows us to determine test sensitivity and specificity that are relevant to the whole population, not just those that are identified as positive or negative by a good but imperfect test.

As described in the previous section, in the absence of a gold standard, we propose to make use of a two-stage approach. In the first stage a small number of animals are selected for testing with the almost perfect test, and the DIVA test. Thereafter we use a latent class approach on animals from two groups: one group being composed of animals selected as being very likely to be uninfected with bTB, the other group being composed of animals that are very likely to have bTB. The selection process for these animals can be thought of as implicitly defining a specificity and sensitivity:

1. A group of putative negative animals. These will be sampled from an area recognized as bTB free. They will have been declared single intradermal comparative tuberculin test (SICTT) negative within the previous 12 months, and they will come from a farm with no animals bought in since that previous test. This selection process for negative animals itself has a specificity of well over 99%. For example, if we consider Scotland, between 2008 and 2013, the percentage of tested farms that have had movement restrictions put in place is consistently at or below 2%^1^,^2^. We can use this figure as an estimate of the probability that we might inadvertently select animals from a farm undergoing a breakdown. Note that this probability is likely to be an overestimate, since the 2% relates to herds that have not undergone testing during the previous 4 years, whereas we would select animals from farms tested in the previous 12 months. Pooling over farms that experienced a breakdown in bTB status between 2002 and 2008, 1.2% of animals were infected with bTB [59 out of 4,958 animals^2^). By combining the probability of selecting a herd undergoing a breakdown with the probability of selecting a positive animal from a breakdown-herd, it follows that buying animals randomly from a number of previously-bTB-free herds might be expected to result ~0.02% of animals being bTB-positive i.e., classing them as negative on the basis of the herd history has an implicit specificity of 99.98%. This is based on sampling a large number of farms, each contributing a small number of animals, resulting in an estimate which will not be overly affected by overdispersion arising from the specific situations on the contributing farms or which needs to be adjusted for the associations we might expect to see between disease status and herd size or farm-specific management factors. In practice, further *post mortem* examination would be used to demonstrate a lack of lesions in these animals, increasing this notional specificity further. Further constraints could be applied to increase the notional specificity of the putatively negative animals if it was thought appropriate (e.g., sourcing only from closed herds).

2. A group of definitively positive animals. These would be SICTT positive, and subsequent to testing with the DIVA test would be subjected to *post mortem* examination. Only the results for those animals with typical lesions identified on *post mortem* and subsequent identification of the causal organism would be retained for calculating the sensitivity. This will ensure that the sensitivity is only calculated using animals that are very likely to have had bTB, and the implicit “gold standard” is assumed therefore to be close to 100%.

## 3. Results

### 3.1. Comparison of calculated sample sizes

If we assume that we have a gold standard test then Tables 2, 3 give the numbers of samples needed to determine the sensitivity or specificity of a test to a given precision at 80% power, using both the “Approximate” and “Exact” methods.

**Table 2:** Table of the numbers of samples needed to determine sensitivity to specified precisions with 95% confidence and 80% power in the presence of a gold standard test.

**Assumed sensitivity (%)**	**Precision (%)**	**Number required (approx. method)**	**Number required (exact method)**
70	±5	323	353
70	±1	8,067	8,230
75	±5	289	320
75	±1	7,203	7,382

**Table 3:** Table of the numbers of samples needed to determine specificity to specified precisions with 95% confidence and 80% power in the presence of a gold standard test.

**Assumed specificity (%)**	**Precision (%)**	**Number required (approx. method)**	**Number required (exact method)**
99.5	±0.5	765	1,226
99.5	±0.2	4,778	5,974
99.85	±0.5	231	696
99.85	±0.2	1,439	2,508

Given our uncertainty about the true sensitivity of the test, there is actually little or no scientifically or logistically relevant difference in the two sets of sample size figures for estimating sensitivity. However, because the assumed values of the specificity are very close to 100%, there are appreciable differences in the estimates arising from the two methods. In this situation, we would recommend use of the exact method.

Evidence from APHA suggests that the DIVA test can have a cutoff set that results in a specificity of 0.999 and a sensitivity of 0.733 (8). From Table 2 we can see that if the sensitivity of the proposed DIVA test is ~75% then use of 300 positive animals will probably allow us to say that the true sensitivity lies between 70 and 80%. From Table 2, if the true specificity is 99.9% then we would require about 1,000 samples to determine that the specificity lies between 99.4 and 100% with a 95% confidence. If we are required to demonstrate cost effectiveness in the UK situation then the previously mentioned threshold of 99.85% applies (9). To demonstrate this using the equations given for the exact method above, we can calculate that we would need at least 20,000 samples. Note that these samples must be independent: it is not appropriate to test multiple samples from a single animal, as these are potentially highly correlated, unless separated widely in time.

### 3.2. Results from no-gold-standard analysis

**Figure 1:**
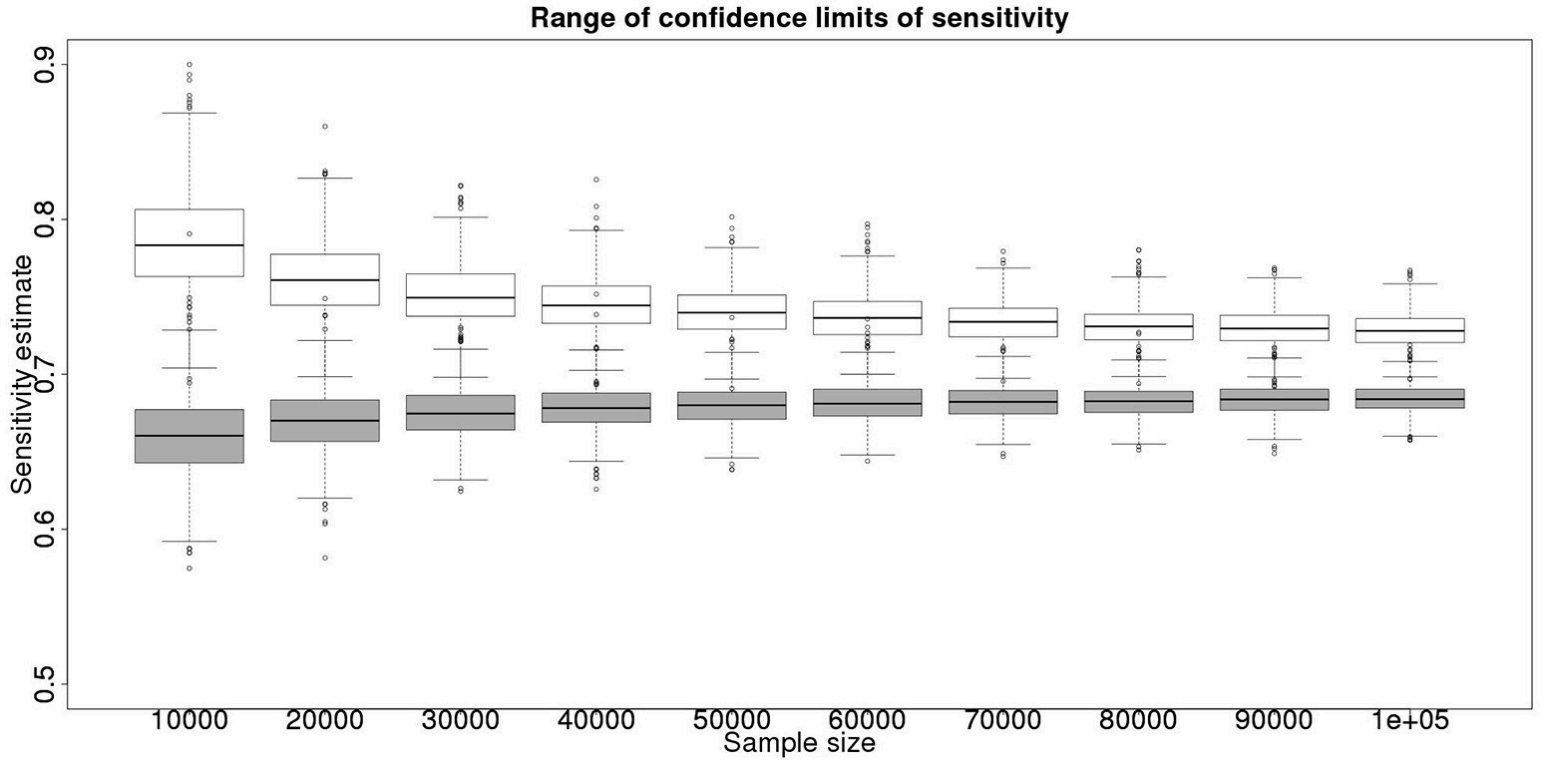
The effect of total sample size on the estimates of the upper and lower 95% confidence limit for a latent class analysis of sensitivity, true value 0.70. The box and whisker plots denote the smallest, first quartile, median, third quartile, and maximum values obtained by analysing 1,000 simulated data sets. Clear box and whisker plots represent the upper confidence limit, gray the lower.

**Figure 2:**
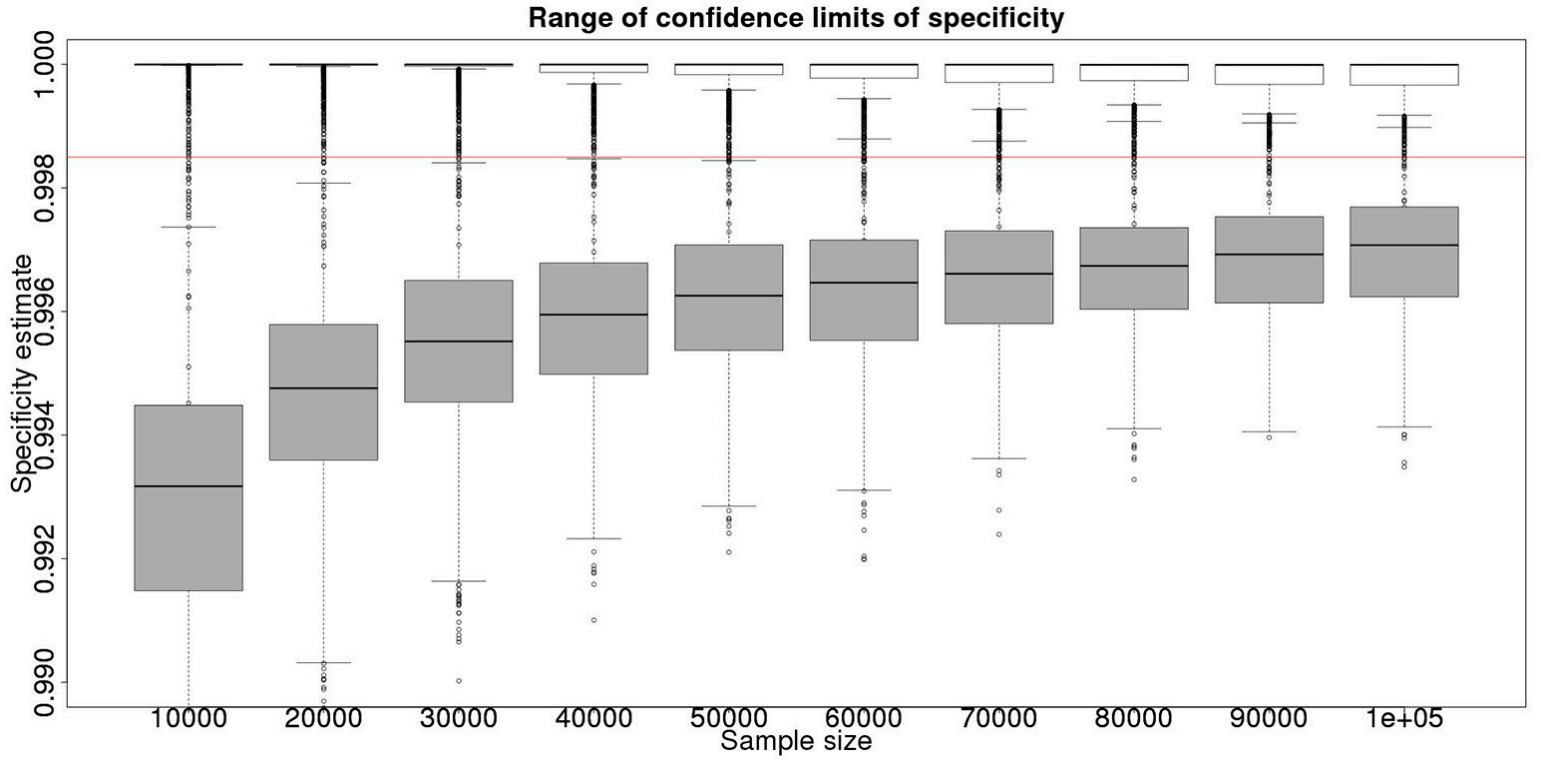
The effect of total sample size on the estimates of the upper and lower 95% confidence limit for a Hui–Walter latent class analysis of specificity, true value 0.999. The box and whisker plots denote the smallest, first quartile, median, third quartile, and maximum values obtained by analysing 1,000 simulated data sets. Clear box and whisker plots represent the upper confidence limit, gray the lower. The red line represents a specificity of 0.9985: the limit for economic equivalence to the present situation, according to the modeling from Conlan et al. (9). See text for details.

In the absence of a gold standard test, the latent class analysis is essential. If we have two populations, of equal size, but dissimilar prevalences, then the precision to which we can determine the test sensitivity for varying total population size is detailed in Figure 1 and for specificity in Figure 2. As can be seen, with 50,000 samples we expect to be able to determine a test with sensitivity of 70% to a precision of about ±5% and a test with specificity of 99.9% with precision of about ±0.4%.

### 3.3. Combined approach

**Figure 3:**
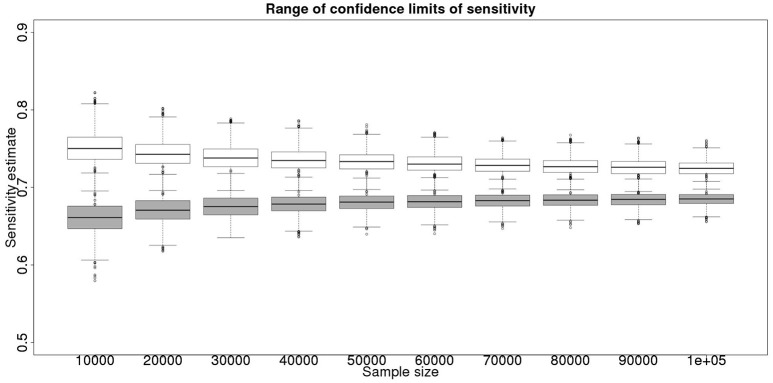
The effect of total sample size on the estimates of the upper and lower 95% confidence limit for a combined analysis of sensitivity, true value 0.70. The box and whisker plots denote the smallest, first quartile, median, third quartile, and maximum values obtained by analysing 1,000 simulated data sets. Clear box and whisker plots represent the upper confidence limit, gray the lower.

**Figure 4:**
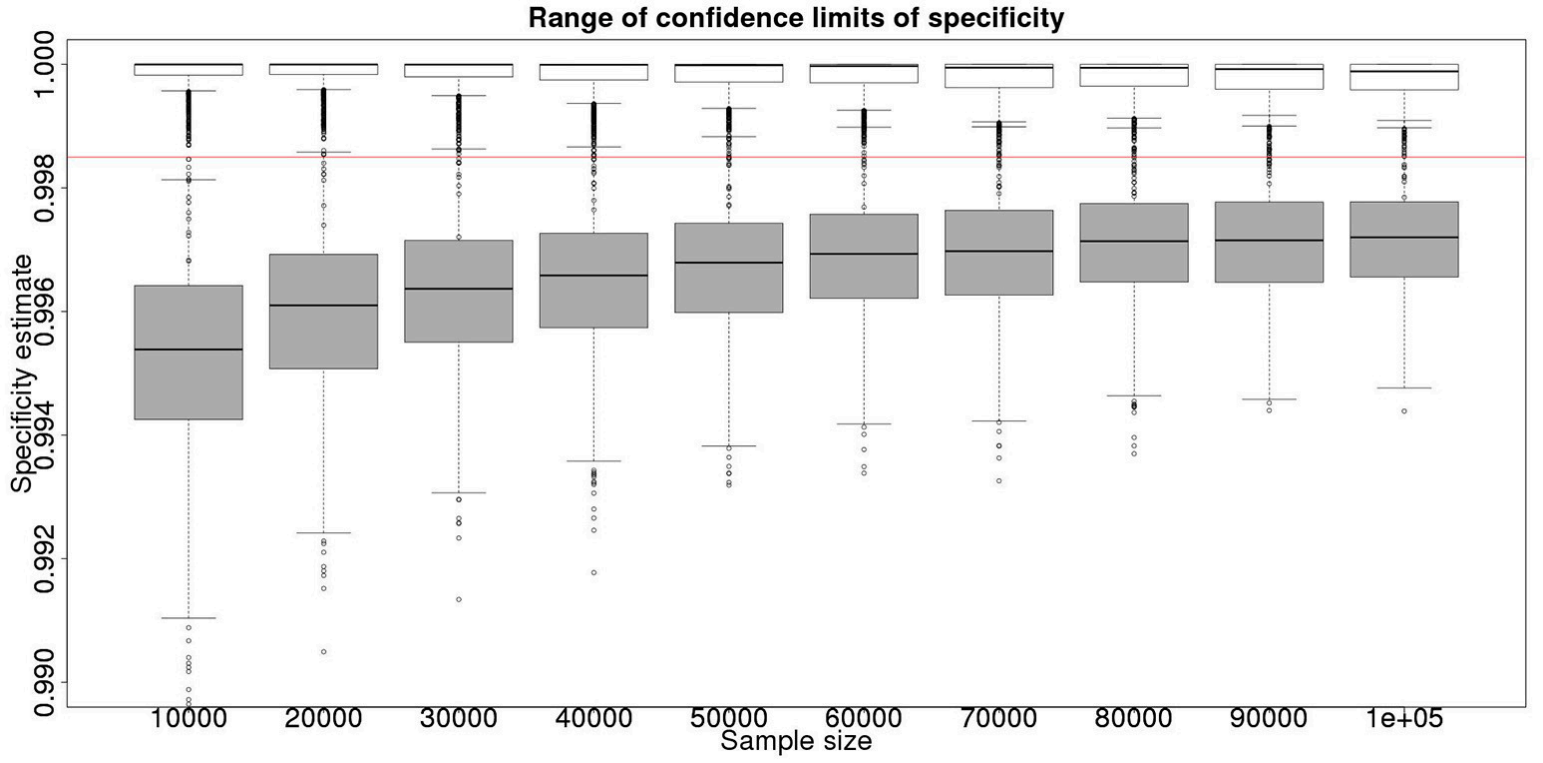
The effect of total sample size on the estimates of the upper and lower 95% confidence limit for a combined analysis of specificity, true value 0.999. The box and whisker plots denote the smallest, first quartile, median, third quartile, and maximum values obtained by analysing 1,000 simulated data sets. Clear box and whisker plots represent the upper confidence limit, gray the lower. The red line represents a specificity of 0.9985: the limit for economic equivalence to the present situation, according to the modeling from Conlan et al. (9).

Although not a true gold standard, *post mortem* identification of lesions with subsequent identification of the causal agent has 100% specificity. If we assume that it represents a perfect test we can use the results from the *post mortem* examination to inform our priors for the latent class analysis. These results, assuming a test with sensitivity of 70% and specificity of 99.9% are detailed in Figure 3 for the sensitivity and Figure 4 for the specificity.

These figures show the median and 95% credible interval for the posterior distribution, and present the distribution of the median or upper and lower limits of the 95% CI across the 1,000 data sample simulations/analyses.

The plots produced from the simulated analyses summarize two important aspects of the effect of increasing sample size. Looking at the distance between the upper and lower limits of the confidence interval, when comparing median to median, first quartile to third quartile, these decrease with increased sample size. This result demonstrates that the larger sample size does tend to reduce the size of the resulting confidence interval for the given sensitivity. Also, looking at the width of the box plots for all the upper limits and all the lower limits for different sample sizes, it is notable that the box plot width tends to shrink with larger samples. This result demonstrates that the uncertainty in the values taken by the confidence limits will also tend to reduce (i.e., results are likely to be more consistent). Hence, the extra information in the combined analysis both decreases the range of the confidence interval somewhat, and decreases the uncertainty in the observed values of the confidence limits. However, in a study with 300 positive animals and 1,000 negative animals the benefit of a combined analysis would be small. It is likely that in any trial some animals, both positive and negative, will be subject to *post mortem* examination and bacterial isolation. Inclusion of these individuals' true status would improve the estimates from the combined analysis. However, given that the number of such animals is likely to be unknown *a priori*, their effect is not included in these calculations.

From Figures 2, 4 it is clear that even with 50,000 samples we would not be able to say with 95% certainty that the specificity is above the 0.9985 limit based on the work of Conlan et al. (9). The results for the lower limit of the 95% CI for these examples is summarized in Table 4. In the combined analysis the precision is greater than the analysis using the latent class method alone, as evidenced by the lower limit of the 95% CI being closer at all quantiles to the simulation value of 0.999. In addition, the uncertainty in the estimate of the lower limit of the 95% CI is lower when using the combined approach rather than the latent class alone, as evidenced by the decrease in it's 90% range. As the sample size increases these effects decrease because the posterior estimate becomes dominated by the new data, rather than the priors derived from the gold standard results.

**Table 4:** Table of the 90%CI for the lower 95% credible limit for the test specificity when using the latent class analysis alone, and when using the combined method outlined in the text, true value 0.999.

**Sample size**	**Latent class**	**Combined method**
1,000	0.9886–0.9945	0.9924–0.9966
2,000	0.9915–0.9958	0.9938–0.9971
3,000	0.9927–0.9965	0.9944–0.9974
4,000	0.9934–0.9968	0.9946–0.9976
5,000	0.9938–0.9971	0.9951–0.9977
6,000	0.9941–0.9972	0.9953–0.9978
7,000	0.9945–0.9973	0.9954–0.9979
8,000	0.9949–0.9974	0.9956–0.9980
9,000	0.9951–0.9975	0.9957–0.9980
10,000	0.9953–0.9977	0.9959–0.9980

*The combined method gives greater precision: on average it is nearer the true value. There is also less uncertainty in the estimate of the lower limit of credible interval, since the range in the table is smaller*.

**Figure 5:**
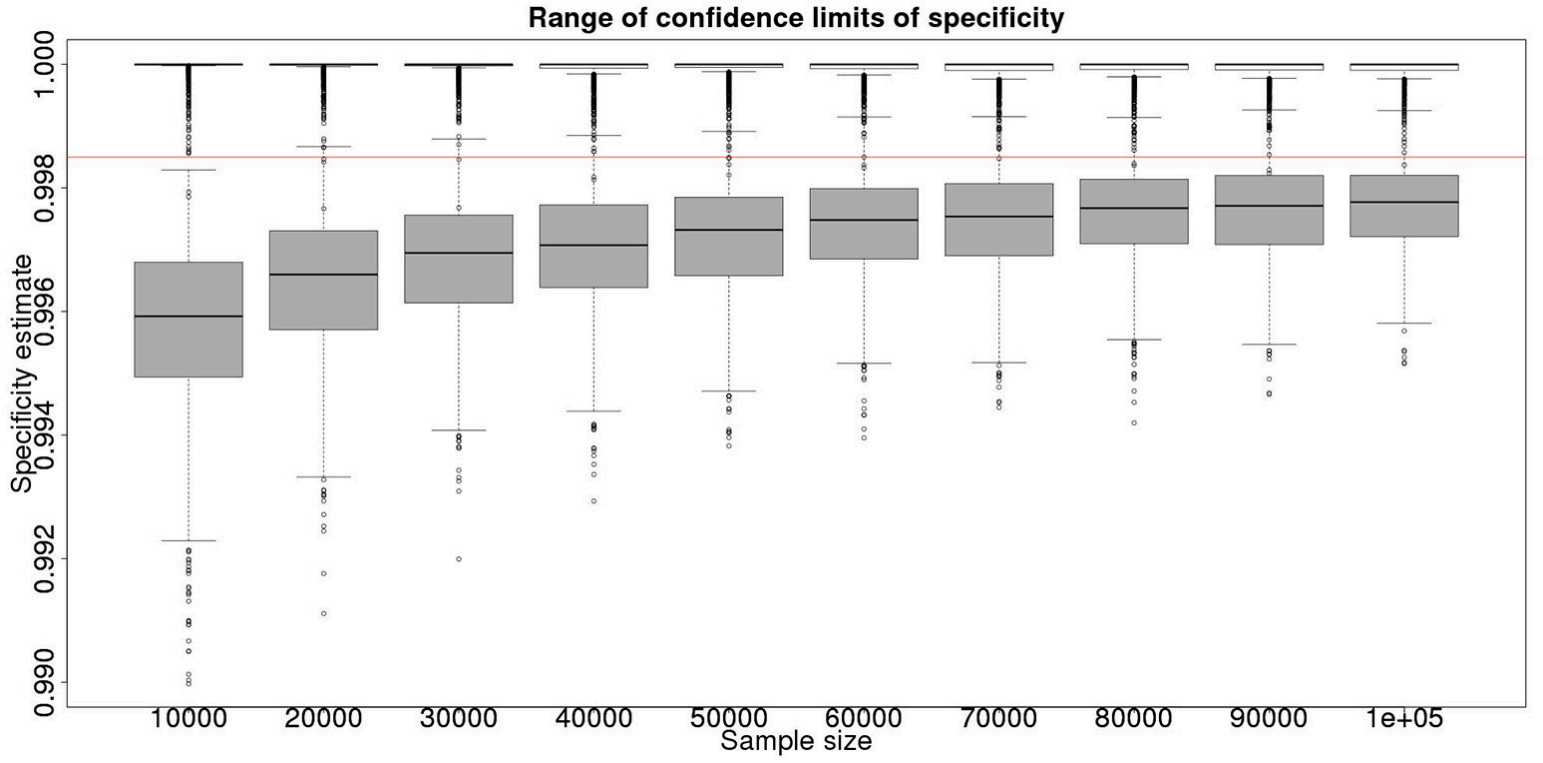
The effect of total sample size on the estimates of the upper and lower 95% confidence limit for a combined analysis of specificity, true value 0.9995. The box and whisker plots denote the smallest, first quartile, median, third quartile, and maximum values obtained by analysing 1,000 simulated data sets. Clear box and whisker plots represent the upper confidence limit, gray the lower. The red line represents a specificity of 0.9985: the limit for economic equivalence to the present situation, according to the modeling from Conlan et al. (9).

These calculations are made on the basis of assuming a true specificity of 0.999, the figure suggested as the true value by APHA (8). If we assume the true value to be the slightly higher value of 0.9995 then the results are presented in Figure 5 show that even then we have only a small chance of saying with 95% certainty that the real value is indeed above the critical threshold of 0.9985.

**Figure 6:**
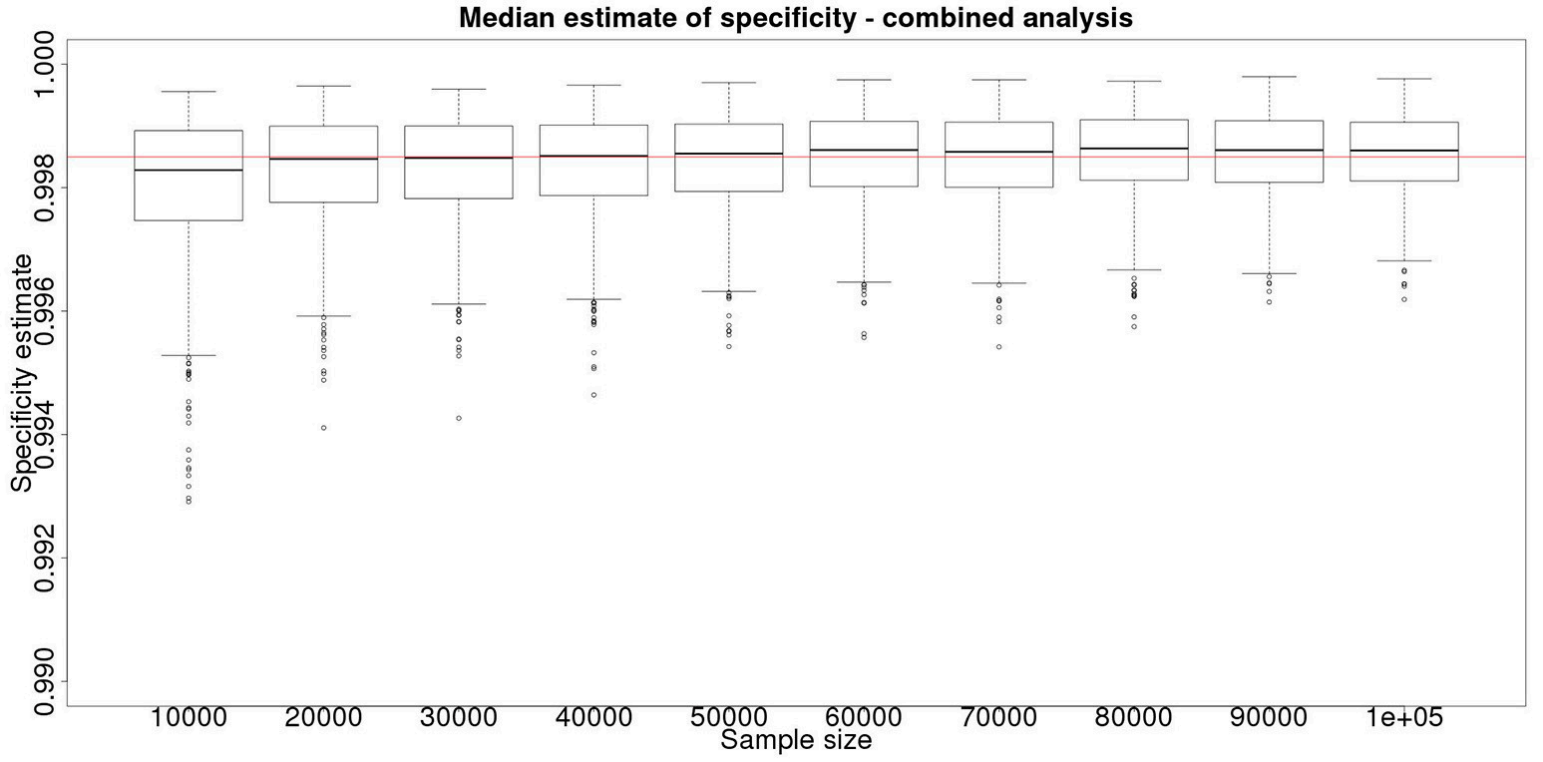
The effect of total sample size on the estimates of the median for a combined analysis of sensitivity, true value 0.999. The box and whisker plots denote the smallest, first quartile, median, third quartile, and maximum values obtained by analysing 1,000 simulated data sets.

However, is this a reasonable approach? Based on their simulation model of bTB in the UK, Conlan et al. (9) state that the true value for the DIVA specificity has to be at or above 0.9985 for there to be no associated increase in the number of culled animals arising from the use of the DIVA test. If the true specificity was in fact 0.9985 then, based on the model results, this test would meet the criterion for introduction. However, a statistical analysis of the data produced by such a test is likely not to be able to support a claim, since randomness in the results and the subsequent uncertainty in the estimates derived from statistical analysis of the results are likely to give rise to a 95% CI that includes values less than the target value. Such an outcome will lead to us being unable to say with reasonable certainty that the true value is not < 0.9985%. A more useful approach is to demonstrate non-inferiority (15), information about which, in the current context, can be thought of as being summarized by the distribution of estimates of the lower credibility interval point. It is implicit in this approach that the true value of the specificity will be greater than the target. Hence, in Figure 6, we present the distribution of the median estimates for the specificity where the true value is 0.999. With 50,000 samples we can say that over 50% of the time we would expect a median in excess of 0.998, and from Figure 5 a lower 95% CI limit >0.9965.

## 4. Discussion

The sensitivity and specificity of a test are arguably the most important measures of the properties and utility of a diagnostic test. However, when these values are quoted it is rare for the precision of the estimates also to be presented. We have demonstrated here that establishing a reasonable precision, particularly when the sensitivity or specificity is high, requires a large sample size, and hence appreciable effort and cost.

The sample size required to estimate sensitivity and specificity to a specific precision is smaller when a gold standard test is available, since the greater knowledge of the properties of the populations gives rise to parameter estimates with lower levels of uncertainty. In the absence of a gold standard, the true status of an individual is unknown and so a latent class analysis is required to obtain estimates of sensitivity and specificity. A combined approach, with a pilot study using a gold standard followed by a no-gold standard Hui–Walter type study can improve the precision of the latent class analysis by using information derived from the pilot study, even if we do not consider the “gold standard” to be perfect.

The latent class approach presented here depends on conditional independence of the two tests. That is, if we knew the true status of an individual then the result of one of the tests depends only on the test sensitivity or specificity, and the result of the other test would give us no extra information to predict this result. In practice, this is rarely the case. However, methods exist to account for this [see, for example (16)]. These methods require the use of more than two populations, since they fit extra parameters to account for the dependence of tests. They also increase the uncertainty in the results further for a given sample size.

The Hui–Walter approach, when extended to incorporate vaccination, has the advantage that we do not need to sample a particular subset of individuals from the population: we do not need to identify animals as positive or negative. Hence, the results of such an analysis are directly relevant to the population from which the samples for the latent class analysis are drawn. Thus, in our case we do not have to rely on observations derived specifically from (for example) “Scottish animals” (i.e., those from a disease-free area) or “animals with *post mortem* signs.” This relaxation of assumptions is desirable, since such animals may not be typical of negative and positive animals respectively.

An additional benefit of using the combined Hui–Walter approach is that an estimate for the efficacy of the vaccine in addition to the properties of the diagnostic tests is available. This is particularly relevant for situations such as the introduction of new bTB vaccines, which may have been evaluated in trials, but where the real-world efficacy is still largely unknown. While the resulting efficacy estimates from the Hui–Walter equations are not identical to those from a study designed for efficacy evaluation, simulations indicate that they would still be able to quantify the effect of vaccination on a population to within ±10 percentage-units. Thus we estimate the overall effect of vaccination at the population level, rather than the usual definition of efficacy which refers to the effect on the individual. Our estimate, therefore includes any “herd immunity” effect that may be present. These results are not presented here, where we concentrate on the estimates of sensitivity and specificity, but are available from the corresponding author.

In general we recommend making an initial assessment of sensitivity and specificity using a small number of known positive animals and negative animals, all vaccinated and all tested with the DIVA test prior to *post mortem* inspection and causal agent identification. This would facilitate an initial analysis to define the sensitivity and specificity with reasonable precision, as specified in section 2.1, confirming that the properties of the diagnostic test were such that it was not inappropriate for the vaccine trial to proceed. Thereafter we recommend employing vaccine trial animals to further increase precision. This would facilitate an analysis to not only make more precise estimates of the sensitivity and specificity (reducing the uncertainty in trial results relating to cost effectiveness, and providing more precise estimates of the properties of the DIVA test), but also to infer an estimate of the efficacy of the vaccine, as defined by the relative decrease in the prevalence of infection in vaccinated as opposed to unvaccinated animals. Since Bayesian analysis does not require us to await all the results being obtained prior to performing the analysis, this approach could be used to perform interim analyses for the estimates of the DIVA test sensitivity and specificity at regular intervals, perhaps annually. This in turn would allow the trial to be stopped early if the evidence suggested that the required efficacy, sensitivity and specificity were unlikely to be reached.

If the vaccine trial population can be divided into two groups with appreciably different disease prevalences, then the combined Hui–Walter method can be used, providing valuable information about the properties of the DIVA test in the vaccinated and unvaccinated populations. If this is not the case, we would be forced to assume that the sensitivity and specificity are the same for both vaccinated and non-vaccinated animals and employ the standard latent class analysis. If this is necessary, and the sensitivities and specificities of the test in the two populations are indeed similar, then with around 40,000 samples we would expect similar results to the values presented here for 80,000 samples, since there would be only half the number of parameters to estimate in the model. If the sensitivities and specificities were not similar, however, the apparent gain in power would be spurious since, by assuming that they are the same in each group the resulting single set of estimates would be biased in opposite directions within the vaccinated and non-vaccinated groups. It is preferable to design any such study on the assumption that we can and will estimate the sensitivities and specificities separately, unless we are sufficiently confident that they are similar across the vaccinated and unvaccinated populations that we do not wish to provide evidence to regulatory agencies about the properties of the test specific to each of the two populations.

Both gold standard and Hui–Walter approaches are valuable and important tools in analysing the properties of diagnostic tests, with their applicability depending on the properties of the available data and any other constraints which might arise in a given situation. However, we believe that the results in this paper have shown that where possible, combining these different approaches makes it possible to utilize some of the advantages of each, and hence enhance the overall analysis.

## Author contributions

GR: programming and running latent class models, production of figures, writing manuscript, commenting on manuscript; GI: initial ideas, project supervision, running latent class models, writing manuscript, commenting on manuscript; IM: initial ideas, implementation of exact method, writing manuscript, commenting on manuscript.

### Conflict of interest statement

The authors declare that the research was conducted in the absence of any commercial or financial relationships that could be construed as a potential conflict of interest.

## Footnotes

^1^bovinetb-scotland-12nov14.xls; 2014. Available online at: https://www.gov.uk/government/uploads/system/uploads/attachment_data/file/372959/bovinetb-scotland-12nov14.xls

^2^Risk-based Surveillance for Tuberculosis in Cattle (bTB) UGW/003/10. (2011). Available online at: http://www.gov.scot/resource/doc/351730/0118086.pdf
